# Basis for Immunotherapy for Treatment of Meningiomas

**DOI:** 10.3389/fneur.2020.00945

**Published:** 2020-08-28

**Authors:** Tomas Garzon-Muvdi, Destiny D. Bailey, Mark N. Pernik, Edward Pan

**Affiliations:** ^1^Department of Neurosurgery, UT Southwestern Medical Center, Dallas, TX, United States; ^2^Department of Neurology, UT Southwestern Medical Center, Dallas, TX, United States

**Keywords:** meningioma, immunotherapy, high grade meningioma, PD-1, checkpoint blockade

## Abstract

Meningiomas are common tumors that account for approximately one third of CNS tumors diagnosed every year. They are classified by the World Health Organization in grades I-III. Higher grades have an increased rate of growth, invasiveness, rate of recurrence, and worse outcomes than lower grades. Most meningiomas are grade I, while ~18% of meningiomas are grade II and III in hospital-based series. Meningiomas are typically “benign” tumors that are treated with surgery and radiation. However, when they recur or are unresectable, treatment options are very limited, especially since they are chemotherapy-resistant. Recent advances in the treatment of cancers with immunotherapy have focused on checkpoint blockade as well as other types of immunotherapy. There is emerging evidence supporting the use of immunotherapy as a potentially effective treatment strategy for meningioma patients. The immune microenvironment of meningiomas is a complex interplay of genetic alterations, immunomodulatory protein expression, and tumor-immune cell interactions. Meningiomas are known to be infiltrated by immune cells including microglia, macrophages, B-cells, and T-cells. Several mechanisms contribute to decreased an ti-tumor immune response, allowing tumor growth and evasion of the immune system. We discuss the most current knowledge on the immune micro-environment of meningiomas, preclinical findings of immunotherapy in meningiomas, meningioma immunotherapy clinical trials, and also offer insight into future prospects for immunotherapies in meningiomas.

## Meningiomas: Epidemiology and Histopathology

Meningiomas are central nervous system (CNS) tumors that arise from arachnoidal cap cells and the dura mater that covers the brain. Meningiomas account for more than one third of CNS tumors and more than one fourth of all intradural extramedullary spinal tumors diagnosed in the United States (1, 2). World Health Organization (WHO) classifies them into three categories: WHO grade I (benign) meningiomas are slow growing and cause neurologic symptoms through compression of adjacent structures; WHO grade II (atypical) and III (anaplastic) meningiomas are more aggressive with significantly increased recurrence rates and worse prognoses.

WHO grade I meningiomas are more frequent in women, whereas the incidence in men is equal or greater in grades II and III meningiomas. The incidence of meningiomas has been increasing in more recent years due to longer life expectancy and more frequent imaging studies being done for a wider range of indications (3). Studies evaluating the epidemiology of meningiomas have given a wide range of results because some of these studies are hospital-based, some are population-based, and some are autopsy-based. In population-based studies, ~5% of meningiomas are atypical and malignant (WHO II and III) (4, 5). Hospital-based studies overestimate the incidence of high-grade meningiomas (HGMs) stating that 6–15% are WHO grade II meningiomas and 2–4% are WHO grade III meningiomas (6, 7). Women more frequently present with WHO grade I or II meningiomas. WHO grade III meningiomas present with the same frequency in men and in women (5).

The criteria of the WHO classification of meningiomas is discussed in Table 1. WHO grade I meningiomas are subdivided into multiple subtypes according to their microscopic appearance. The WHO criteria define grade I meningiomas as having <4 mitoses per 10 high-power fields (hpf) and no evidence of histological variance. WHO grade II meningiomas are defined as having increased mitosis (4–19 per 10 hpf), evidence of a histological variant (chordoid or clear cell), or three of five of the following criteria: spontaneous necrosis, sheeting, prominent nucleoli, high cellularity, or small cells. The WHO classification was revised in 2007 to include brain invasion as a criterion for the diagnosis of WHO grade II meningioma (10). They are associated with a 10-year progression-free survival of 26% without radiation therapy and 45% with radiation therapy (11). WHO grade III meningiomas are very aggressive tumors. The criteria include increased mitosis (>20 per 10 hpf), true anaplasia, and papillary and rhabdoid tumors. These tumors are more likely to recur after resection compared to WHO grade I meningiomas. In different series, the rates of recurrence of WHO grade I, II, and III tumors range from 7–25%, 29–59%, and 50–94%, respectively (12–17).

**Table 1:** Classification of meningiomas.

**Subtype of meningioma**	**WHO grade**	**Demographics**	**Pathology criteria**	**Progression-free survival/survival at 10 years**
Psammomatous	I	Women > Men Age at Dx: 50–60 years of age	Mitoses <4 in 10 HPF MIB mean 3.8%	75–87%/79–90%
Angiomatous	I			
Transitional	I			
Meningothelial	I			
Fibrous	I			
Microcystic	I			
Secretory	I			
Metaplastic	I			
Lymphoplasmacyte-rich	I			
Atypical	II	Women = or > Men Age at Dx: 40–50 years of age	Mitoses 4–19 in 10 HPF 3 of 5: Spontaneous necrosis Sheeting Prominent nucleoli High cellularity Small cells OR brain invasion MIB mean 7.2%	25–80%/50–70%
Chordoid	II			
Clear cell	II			
Rhabdoid	III	Men > Women Age at Dx: 40-50 years of age	Mitoses >20 in 10 HPF Obvious anaplasia Papillary or rhabdoid histology MIB mean 15%	<5%/15–40%
Papillary	III			
Anaplastic/malignant	III			

*Classification of meningiomas according to the World Health Organization. Demographic variables and clinical outcomes (8, 9)*.

## Prognostic Factors for Meningiomas

Several factors independently predict recurrence-free survival and overall survival in meningiomas outside of WHO criteria. Extent of initial resection is one of the most long-standing and important risk factors for recurrence (18). Even in modern cohorts with extended follow-up, Simpson Grade 1 resections more than double the chance of progression-free survival, especially in high-grade meningiomas (19). In addition to Simpson grade, higher initial tumor size and MIB-1/Ki-67 indices, as well as presence of necrosis may decrease progression- and recurrence-free survival (19–22).

Recent studies have focused on molecular and genome-associated prognostic markers in meningioma (23, 24). Sahm et al. devised a DNA methylation-based grading system for meningioma that accurately stratified risk of recurrence in meningiomas more accurately than current WHO grading by analyzing methylation patterns in 40 genes known to contribute to meningioma genesis and progression (23). Similarly, Katz et al. also demonstrate that methylation patterns on the H3 histone could predict recurrence of WHO grade I and II meningiomas (25). One recent study highlighted the use of RNA sequencing and whole-exome sequencing to predict recurrence more accurately than current WHO criteria (24). Interestingly, in all these studies, the authors report that their methods accurately predicted recurrence in a significant subset of WHO Grade I tumors where recurrence is typically low (23–25). Studies with larger sample sizes are needed to assess how tumor microenvironment and immune infiltrate impact meningioma recurrence and progression.

## Genetic Alterations of Meningiomas

Well-known genetic alterations of meningiomas include monosomy 22 and inactivating mutations of NF2 gene that produces neurofibromin (also known as merlin), as seen in patients with Neurofibromatosis type 2 that develop multiple meningiomas. This alteration has also been identified in a significant proportion of sporadic meningiomas. One study found in primary, non-NF2 mutated meningiomas, the pro-tumor inflammatory mediator IL-1β induced methylation of the NF2 promotor through various mediators that could act as novel targets (26). Other mutations like TRAF7, AKT1, KLF4, SMO, and PIK3CA were more recently described (27, 28) and may represent therapeutic targets (29–31). In one particular study, grade I meningiomas harboring AKT1 mutations had predominantly M2-subtype infiltrating macrophages, indicating a locally immunosuppressed tumor microenvironment (32). Additionally, it has been found that tumors initially diagnosed as WHO grade II meningiomas commonly have loss of NF2 with alterations in SMARCB1 and have an increased H3K27 methylation (33). Recently, PD-L1 levels in tumors with TRAF7 mutations were significantly higher than tumors without TRAF7 alterations, suggesting that patients with TRAF7 mutations could benefit from immunotherapy (34).

Genetic alterations are extremely important in the context of immunotherapy to treat meningiomas for multiple reasons. It is known that tumors with high rates of somatic mutations have a higher load of neoantigens expressed in MHC I molecules in the cellular surface that can elicit a robust anti-tumor immune response. This is true for tumors like melanoma and lung cancer, where mutational burden is correlated with response to immunotherapy (35–38). This is also true for CNS tumors like glioblastoma multiforme (GBM), where only a small subset of GBMs are likely to benefit from checkpoint inhibition (39). Meningiomas are not characterized by a high mutational burden, but there is a subset with higher rates of somatic mutations that may be better candidates for immunotherapy (27, 40). For example, meningiomas commonly have isolated monosomy 22/del(22q) mutations, which show increased tumor-infiltrating M1-subtype macrophages, NK cells, and T-cells (41, 42). Another important aspect of genetic alterations in meningiomas is that molecular subtypes characterized by specific genetic mutations express different checkpoint molecules (34, 43). It is conceivable that by molecularly classifying meningiomas to determine the presence of different mutations, chromosomal derangements, and their relationship to immune cell infiltration, we will be able to determine optimal immunotherapeutic strategies for patients.

## Current Management Options for Meningiomas

The mainstay of treatment for meningiomas is maximal safe surgical resection. If complete resection of the tumor and associated dura is possible, the risk of recurrence decreases significantly. The anatomic location of the meningioma influences the ability to resect the lesion entirely with wide dural margins. Complete resection may not be possible in some cases such as lesions in the skull base, involving the dural venous sinuses, or higher-grade spinal meningiomas with ventral attachment (44). Thus, lesions of the sphenoid wing in the skull base have higher rates of recurrence than convexity lesions, followed by parasagittal lesions.

Even in cases of complete surgical resection, a subset of meningiomas recurs and exhibits aggressive behavior. Recurrences are often difficult to treat and involve important anatomical structures. In these cases, treatment after surgery typically includes fractionated stereotactic radiation (FSR) or stereotactic radiosurgery (SRS). In RTOG 0539, meningiomas were stratified into low risk [grade I and gross total resection (GTR) or subtotal resection (STR)], intermediate risk (recurrent grade I or grade II after GTR), and high risk (STR or recurrent grade II and any grade III). Low risk tumors demonstrated a progression free survival (PFS) of 92%, intermediate risk PFS of 94%, and high risk PFS of 59% at 3 years, respectively (45). There is also a role for SRS to treat low grade meningiomas and previously radiated meningiomas to improve PFS (45, 46). In a recent review of the literature evaluating meningioma recurrence after surgery, recurrence rates ranged from 0–22.5% at 5 years for grade I meningiomas, 15% at 2 years, and 37% of patients over 10 years for grade II meningiomas (47). PFS and overall survival (OS) rates of patients with grade III meningiomas has been reported to range from 0–57% and 33–61% at 5 years, respectively (46, 48, 49). The recurrence-free survival is 12 years for WHO grade I meningiomas, <7 years for grade II meningiomas, and <2.5 years for grade III meningiomas (50). Other factors that predict higher risk of recurrence include high expression of vascular endothelial growth factor (VEGF) and high MIB-1/Ki-67 labeling (51).

Chemotherapy for grade II and grade III meningiomas has not had much success (52). Specific chemotherapeutic agents such as doxorubicin, irinotecan, vincristine, and temozolomide have been evaluated and found to have little efficacy (53). There have been trials testing the utility of receptor tyrosine kinase inhibitors such as gefitinib, erlotinib, and imatinib that target platelet derived and epidermal growth factor receptors. However, these agents have also not been effective against grade II and III meningiomas (54–56). The first randomized control trial for high-grade meningiomas using Trabectedin did not show any survival benefit, indicating that targeted alkylating agents may not be beneficial (57). Antiangiogenic agents that target the VEGF receptor have also been evaluated and found to have suboptimal results (58, 59). Genetic alterations such as mutations in AKT1, PIK3CA, SMO, and NF2 are being targeted in a phase II trial using vismodegib and FAK inhibitor GSK2256098 in trial NCT02523014. Other genetic alterations that are treatment targets have been tested in clinical trials (60).

## Immunotherapeutic Strategies for High Grade Meningiomas

Conventional therapeutic modalities (including radiation, chemotherapy, and targeted medical therapies) have not been effective in improving progression-free survival for HGMs. Therefore, new ways to treat these aggressive tumors are necessary. There are several immunotherapeutic agents that leverage the host immune system against disease, including checkpoint inhibitors, chimeric antigen receptor T cells (CAR-T), monoclonal antibodies, oncolytic viruses, and cytokines. This review focuses on strategies prevalent in existing literature such as checkpoint inhibition (CI) and CAR-T cells which have been used in studies to leverage the immune system against meningiomas.

Immunotherapy, particularly CI, has shown improved survival in other solid tumors such as lung cancer and melanoma patients, in addition to many other types of cancer (61–69). Considerable interest and effort has been placed in treating brain tumor patients with immunotherapy, particularly GBM. A recent clinical trial using anti-PD-1 to treat patients with recurrent glioblastoma, Checkmate-143, had objective responses in only 8% of study patients (70). The low response to immunotherapy may be due to factors such as the tumor stroma physically limiting the entry of immune cells, low mutational burden from low amounts of tumor antigens, and even conventional therapies like chemotherapy that deplete the immune system (71, 72). The ability of tumors to escape immune surveillance is a key step in oncogenesis (73).

In light of the evidence presented above, immunotherapy becomes a potentially attractive alternative for HGM patients. It has the potential to overcome the limitations of surgical, radiotherapeutic, and chemotherapeutic treatment strategies, as immune cells have the ability to infiltrate tumor tissues and target individual cells with anti-tumor capability. It has been shown that immune cell infiltration is an important indicator of prognosis in different tumors (74–77). Furthermore, the composition of the immune infiltrate is important because it delineates the character of the immune microenvironment. In other instances, tumors have been classified as “hot” if they respond to immunotherapy and “cold” if they lack a response after immunotherapy. For example, glioblastoma (GBM) is considered a cold tumor because of its lack of response to immunotherapy (70).

Several recent studies have aimed to characterize the interactions between meningiomas and the immune system. Specifically, studies of the immune microenvironment in meningiomas have revealed that checkpoint molecules like NY-ESO-1, PD-L1, PD-L2, B7-H3, and CTLA-4 are expressed in meningiomas and may be at least partly responsible for the suppression of the anti-tumor immune response (43, 78–81). Programmed Death Ligand-1 (PD-L1) is expressed in meningiomas, and expression levels are higher for higher-grade tumors (80). The expression of these proteins has been associated with tumor progression, recurrence, and poor survival outcomes. Additionally, it is clear that meningiomas are infiltrated by T-cells, B-cells, macrophages, and plasma cells (82–85). Fang et al. extensively characterized the immune infiltrate in meningiomas and found that the immune cells infiltrating meningiomas are mainly antigen-experienced T cells and B cells (85). In their study, B cells were activated and underwent immunoglobulin class switching, somatic hypermutation, and clonal expansion. T-cells demonstrated evidence of antigen exposure and increased expression of checkpoint molecules programmed death-1 (PD-1) and T-cell Ig and mucin protein-3 (TIM-3), which can also be a sign of an exhausted phenotype. Tumor-infiltrating lymphocytes in meningiomas are mainly T-cells. Interestingly in WHO grade III meningiomas, the number of CD4 and CD8 T-cells is low and at the same time, the proportion of infiltrating regulatory T-cells (Tregs) is increased (79). These data support the notion that there is an immunosuppressive microenvironment in meningiomas that may contribute to tumor progression.

## Tumor Immune Microenvironment

**Figure 1:**
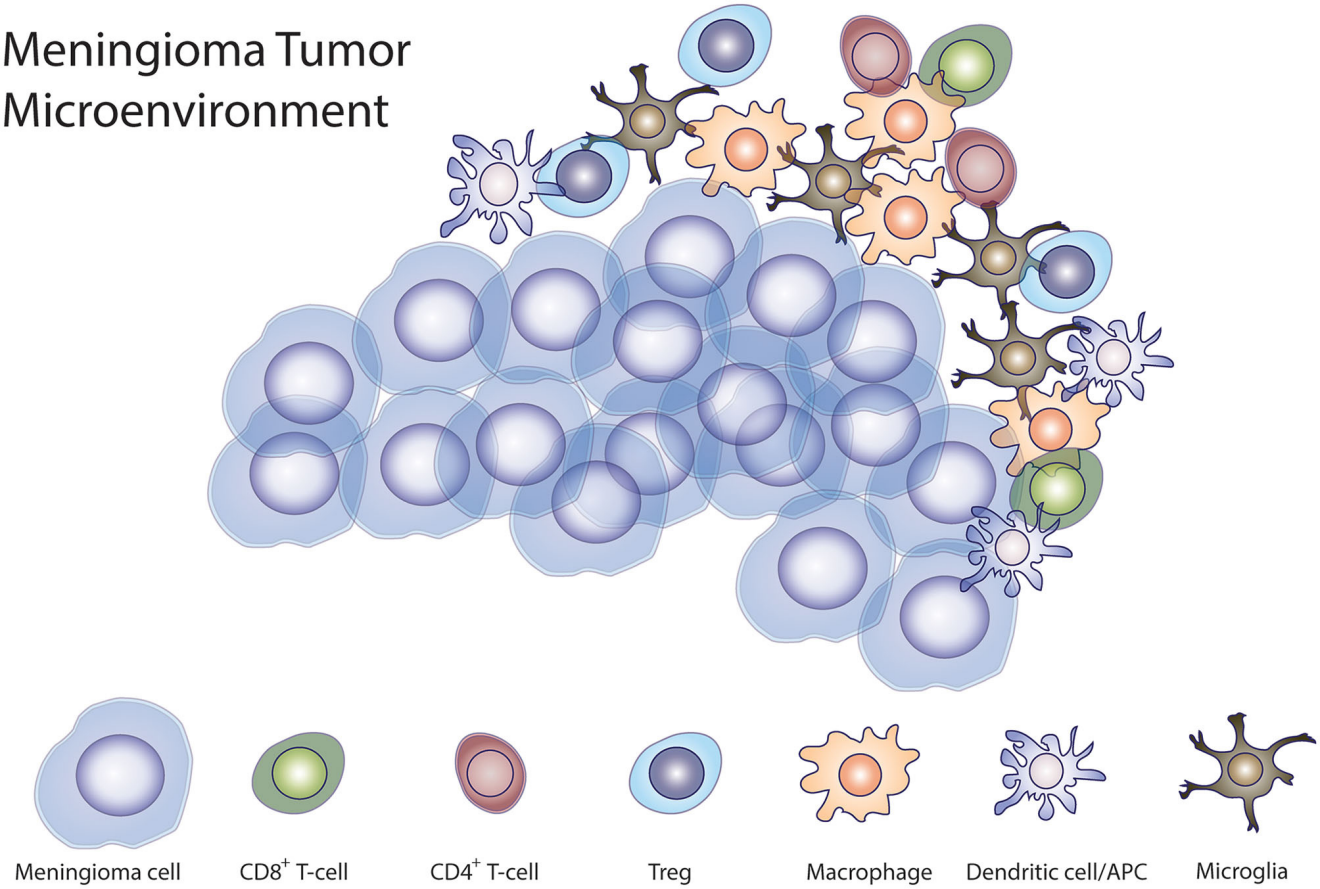
Meningioma immune microenvironment. Illustration demonstrating the interaction of tumor cells with different immune cells demonstrated to be present in the tumor microenvironment in meningiomas.

Meningiomas are not limited by the blood-brain barrier (BBB), making these tumors accessible to the peripheral immune cells. The composition of immune infiltrate in the tumor microenvironment is closely associated with tumor progression (86). A large proportion of cells in the tumor microenvironment in meningiomas are CD45^+^ immune cells. These cells include myeloid cells, CD3^+^ T cells, most of which are CD8^+^ cells, and natural killer (NK) cells (85). In lesser proportion, meningiomas are infiltrated by Tregs, while B cells also infiltrate meningiomas, albeit in lower numbers (85). Macrophages and other myeloid cells infiltrate meningiomas and can acquire immunosuppressive phenotypes (87). Macrophage infiltrate makes up the largest compartment—~18%—of meningiomas and their number increases as the grade of the tumor increases (88). Myeloid derived suppressor cells (MDSCs) have also been isolated from meningiomas. Remarkably, MDSCs are expanded in HGM compared to WHO grade I meningiomas (89). The majority of macrophages that infiltrate meningiomas are polarized to an M2 suppressive phenotype. However, meningiomas harboring a chromosome 22q deletion having a predominance of M1-phase macrophages (90). Macrophages and microglia are recruited to the brain-tumor interface more frequently in WHO grade II and III than in WHO grade I meningiomas (91). Han et al. showed that patients with meningiomas that are infiltrated by PD-L1-expressing macrophages had a worse prognosis (80). CD8^+^ T cells are found in greater quantity in the meningioma microenvironment than other lymphocyte infiltrates (88, 92). The majority of infiltrating T-cells in high-grade meningiomas appear to be exhausted PD1^+^ T cells and T regs (85, 92). Patients with a higher proportion of tumor-infiltrating PD1^+^ T cells have significantly shorter PFS (92). Interestingly, WHO grade III meningiomas have a significantly higher proportion of infiltration of Tregs compared to WHO grade I or II meningiomas (92). B-cells are the least numerous tumor-infiltrating lymphocytes in meningiomas. More B-cells were activated and exposed to antigen (85). There is also evidence that HGMs are able to influence the immune system systemically, given that patients with WHO grade III meningiomas have significantly higher levels of circulating myeloid derived suppressor cells (MDSCs) than WHO grade I and II meningioma patients, suggesting a state of systemic immunosuppression in these patients (92) (Figure 1).

Additionally, the NF2 gene may have implications for the tumor microenvironment (26, 93). Wang et al. find that a proportion of NF2-associated meningiomas expressed PD-L1 on tumoral cells and 100% had tumor-infiltrating lymphocytes (93). In combination with the predominance of M1 pro-inflammatory macrophage infiltrate in meningiomas with 22q deletions, CI could be more efficacious in meningiomas with NF2 inactivation or deletions (42).

## Mechanisms Used to Evade the Immune System

The ability to evade the immune system is an important event in cancer pathophysiology. Expression of ligands on tumor cells to cognate receptors on immune effector cells, such as checkpoint molecules, is an important mechanism used by tumors in general to evade immune anti-tumor response, to limit the effector function of immune cells, and to manipulate immune cells into suppressive tumor-associated cells. Immune cells such as macrophages and lymphocytes bind to these ligands or checkpoint molecules and become quiescent and sometimes immunosuppressive, leaving tumor cells to proliferate unchecked. Checkpoint inhibitor therapy targeted at immune cells associated with checkpoint proteins, like PD-L1, PD-L2, B7-H3, and CTLA-4, could help disinhibit the host immune system to help combat tumor survival and growth (43, 80, 92, 94).

**Figure 2:**
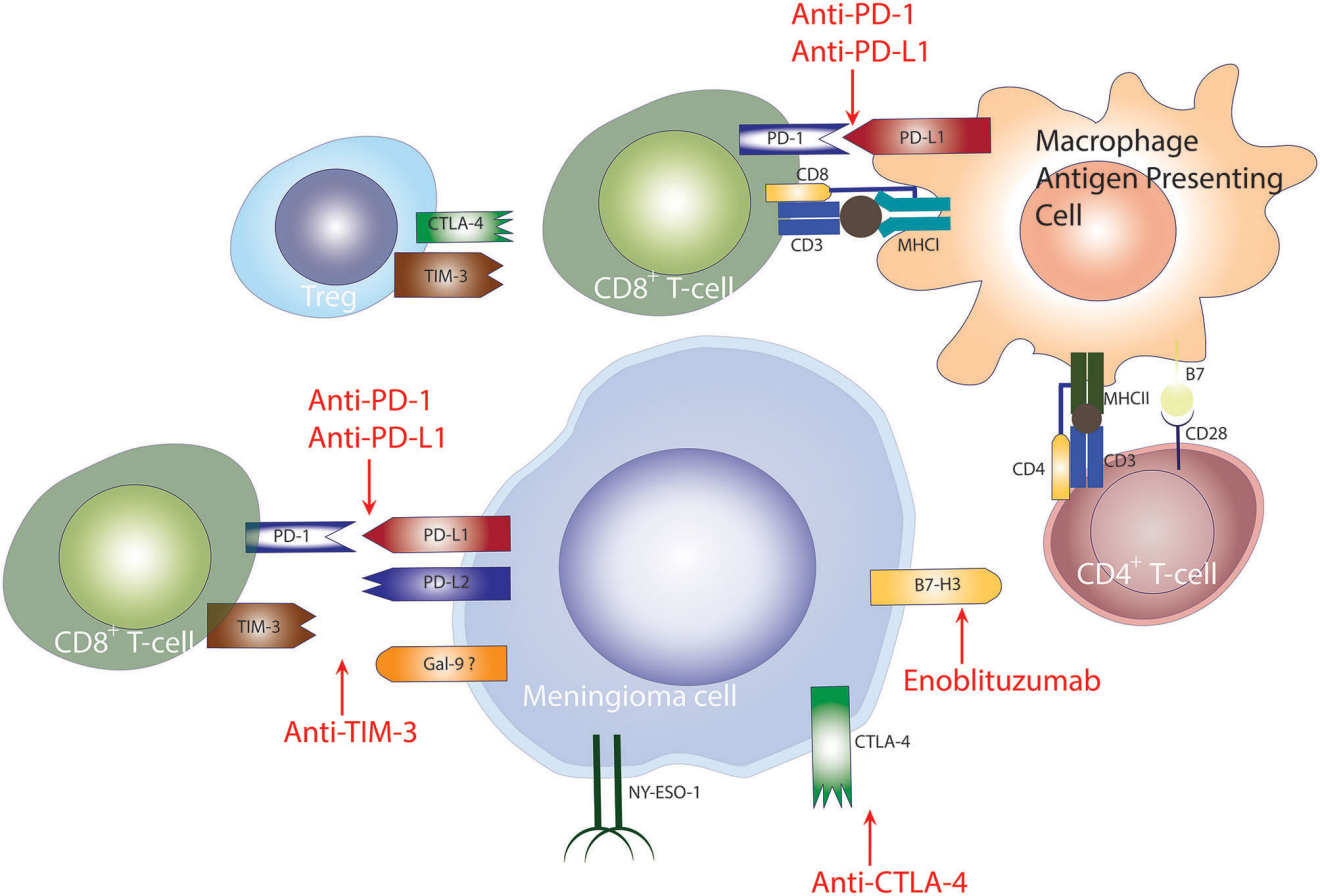
Checkpoint inhibition to treat meningiomas. Checkpoint-mediated mechanisms employed by meningiomas for evasion of the anti-tumor immune response. PD-1 and CTLA-4 signaling diminish T-cell mediated anti-tumor immune response, hampering tumor elimination. Checkpoint blockade of PD-1, CTLA-4, TIM-3 has shown promising results in other cancers.

PD-L1 is expressed on the surface of tumor cells and is a major mechanism used by meningiomas to evade the immune system. PD-L1-expressing tumor cells inhibit T-cell activation by binding to the PD-1 surface receptor on T- and B-cells (94). In meningiomas, the expression of PD-L1 in meningiomas correlates with tumor grade where higher grade tumors demonstrate higher expression of PD-L1 in tumor cells (79, 80, 92). In a study by Han et al. PD-L1 expression was associated with poor survival outcomes in patients with high-grade meningiomas (80), however, other studies have not found an association between PD-L1 expression and PFS, potentially because of the limited number of HGMs in those studies (79, 92). Taken together the data demonstrating increased expression of PD-L1 and the presence of infiltrating PD1^+^ T cells, it is conceivable that PD1 blockade to overcome immunosuppression in high-grade meningiomas may represent a viable and successful treatment strategy to treat patients with high-grade meningiomas (Figure 2).

**Figure 3:**
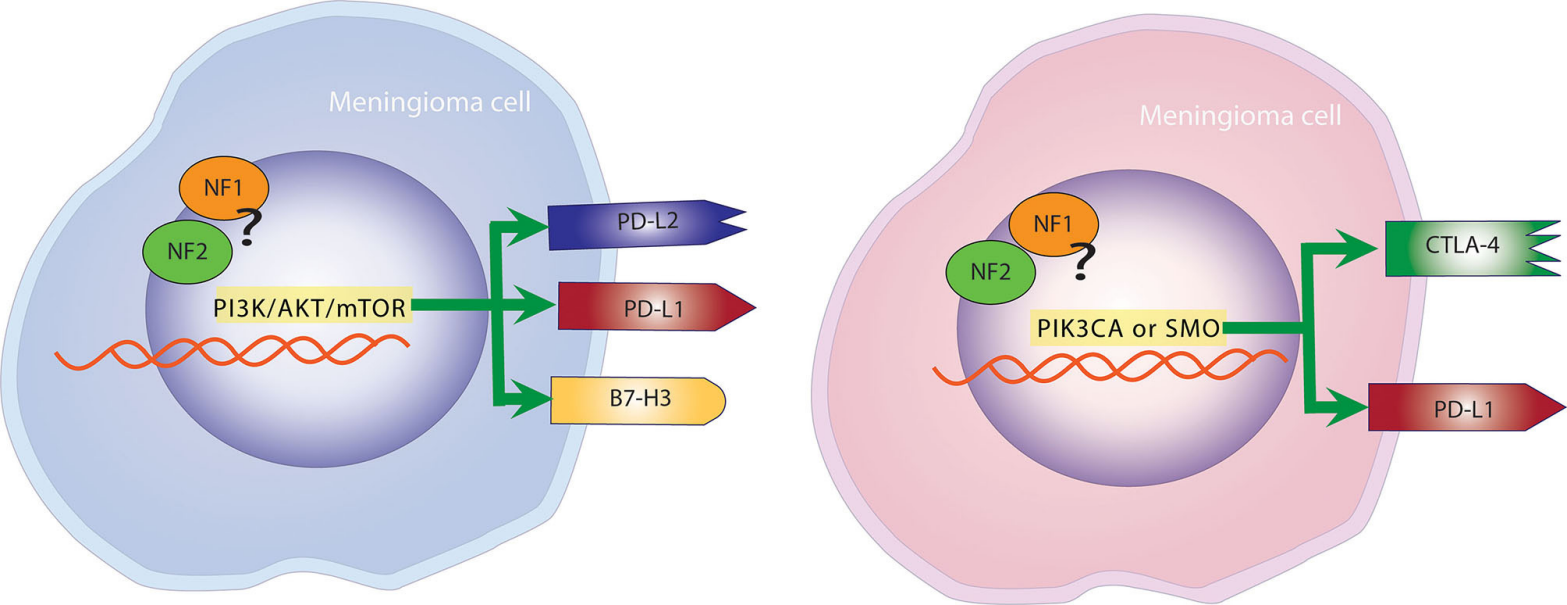
Mutational landscape determines the expression of checkpoint molecules. Left panel: Meningiomas with mutations in the PI3K, AKT, mTOR pathway are more likely to overexpress PD-L1, PD-L2, and B7-H3. Right panel: Meningiomas with alterations in PIK3CA and SMO are more likely to overexpress PD-L1 and CTLA-4.

In a study by Proctor et al. where tumors where genetically characterized, the expression of checkpoint molecules was studied and correlated to specific molecular subtypes of meningiomas (43). PD-L2 and B7-H3 were found to be expressed on the surface of tumor cells at significantly higher levels than PD-L1 and CTLA-4 in Grade I and II meningiomas. Additionally, PD-L2 and B7-H3 were consistently expressed in tumors with mutations in the PI3K/AKT/mTOR pathway, whereas CTLA-4 was found more frequently in tumors with PIK3CA or SMO mutations (43). This finding was particularly important because it ties immune checkpoint molecules to meningiomas regardless of grade (Figure 3).

## Leveraging Immune Microenvironment in Meningioma Treatment

There is significant evidence that supports the use of immunotherapy to treat patients with meningioma, particularly WHO grade II and III meningiomas. In light of these recent discoveries of checkpoint molecule expression and immune infiltrate in the meningioma tumor microenvironment checkpoint blockade is a perfectly logical option. Additionally, compared to other CNS tumors such as GBM, meningiomas are not guarded by the BBB.

### PD1/PD-L1 Checkpoint Blockade in Recurrent Meningiomas

The main avenue of investigation is focused on utilizing monoclonal antibodies targeting PD1/PD-L1 to augment the anti-tumor immune response (34, 95–97). As discussed previously, HGMs have higher levels of expression of PD-L1. Patients with WHO grade III meningiomas also display increased peripheral MDSCs that express PD-L1, suggesting that this contributes to systemic immunosuppression (92). Tumors that have received prior radiation therapy also have significantly higher expression of PD-L1 (80). In light if these data, checkpoint blockade presents itself as a potentially successful strategy to treat high-grade and recurrent tumors. In a mouse model of meningioma, infusion of anti-PD1 antibody avelumab plus highly-active NK cells (HaNK) led to increased survival, showing the importance of innate NK cell activity (97). Currently there are two case reports on PD-L1 checkpoint inhibition for recurrent meningiomas (95, 96). The cases report disease-free recurrence for >2 years in one patient and >6 months in another patient, with both having reductions in tumor volume, cerebral edema, and patient-reported symptoms following nivolumab treatment (95, 96). In contrast to this report, Abele et al. report the development of an atypical grade II meningioma during nivolumab treatment for metastatic renal cell carcinoma in which ~10% of meningioma cells were PD-L1 positive (98). These cases highlight that response to immunotherapy may not be solely dependent on PD-L1 or may be dependent on higher levels of expression.

Based on the existing evidence on PD-L1 expression in recurrent meningiomas, five clinical trials are enrolling patients with to receive anti-PD1 antibodies nivolumab, avelumab, or pembrolizumab (Table 2). An ongoing phase II trial is designed to compare nivolumab alone to combination therapy with the anti-CTLA-4 antibody ipilimumab (NCT02648997). A phase Ib trial will investigate the preoperative use of avelumab in combination with hypo-fractionated proton radiotherapy for 3 months to evaluate its effect on the size of unresected meningiomas (NCT03267836). The other trials are recruiting patients with recurrent HGMs to receive adjuvant immunotherapy as PD1 blockade. These trials represent an opportunity for patients who have undergone multiple resections and radiation treatments with continued recurrence. While results of checkpoint inhibition targeting PD-L1/PD-1 for recurrent meningiomas has been initially positive, there are questions remaining. For instance, despite the association with higher PD-L1 expression in meningioma grade II and III tumors, it is not yet clear whether level of expression will correlate with treatment response (79, 80, 92, 97). It is clear that there is variability even among tumors of the same WHO grade; however, molecular characterization may be used as a biomarker in conjunction with expression of checkpoint molecules to guide the treatment strategy to optimize responses.

**Table 2:** Clinical trials with immunotherapy for meningiomas.

**Study title**	**Drug(s)**	**Phase**	**Estimated enrollment**	**Study sites**	**Primary completion date**	**Trial registration number**
An open-label phase II study of nivolumab in adult participants with recurrent high-grade meningioma	Nivolumab ± Ipilimumab	II	50	Dana-Farber Cancer Institute	February 2020	NCT02648997
A trial of pembrolizumab for refractory atypical and anaplastic meningioma	Pembrolizomab	II	25	Rabin Medical Center	February 2020	NCT03016091
Neoadjuvant avelumab and hypofractionated proton radiation therapy followed by surgery for recurrent radiation-refractory meningioma	Avelumab	Ib	12	Washington University School of Medicine	July 2020	NCT03267836
Phase II trial of pembrolizumab in recurrent or residual high grade meningioma	Pembrolizumab	II	26	Massachusetts General Hospital	March 2021	NCT03279692
Immune checkpoint inhibitor nivolumab in people with select rare CNS cancers	Nivolumab	II	180	National Cancer Institute	December 2020	NCT03173950

### Potential Alternative Targets for Meningioma Immunotherapy

While initial checkpoint inhibition results via PD-1/PD-L1 blockade are promising, other potential targets exist and warrant additional investigation. In the past few years, previously unrecognized immunomodulatory proteins that are highly expressed in meningiomas have been identified. These proteins include PD-L2, B7-H3, CTLA-4, and NY-ESO-1 (43, 78, 80). Recently, a thorough study linking genetic alterations analyzed the expression of three checkpoint molecules previously unidentified in meningiomas including CTLA-4, B7-H3, and PD-L2 (43). B7-H3 and PD-L2 expression levels were significantly higher in patients with genetic mutations in the PI3K/AKT/mTOR pathway, whereas CTLA-4 expression was higher in tumors with mutations in PIK3CA or SMO. Expression of B7-H3 was high in their series of tested tumors, with all 22 of the specimens testing positive, and many showing nearly 100% tumor cell expression (43). While the exact mechanism of B7-H3 interactions are unknown, its blockade has shown promise in reducing tumor growth, while enoblituzumab (anti-B7-H3) is currently being tested in clinical trials for other tumors, including gliomas (43, 99). Given its near ubiquitous expression in meningioma, its potential for a therapeutic target is promising.

Interestingly Proctor et al. found that PD-L2, a receptor for PD-1, was expressed at higher levels compared to PD-L1 throughout all meningioma grades (43). In a series of head and heck squamous cell carcinomas, PD-L2 expression, regardless of PD-L1 expression, predicted responses to pembrolizumab (100). If recapitulated in the current meningioma trials, PD-L2 could play an important role as a biomarker to predict responses to immunotherapy. CTLA-4 is also a potentially important target for immunotherapy as evidenced by its expression in a subset of meningiomas and is currently being tested in combination with PD1 blockade in clinical trial NCT02648997 (43).

Baia et al. also evaluated NY-ESO-1 as also a potential target for immunotherapy. NY-ESO-1 has been demonstrated in cancers aside from meningioma. This molecule has the ability to provoke humoral and cellular immune responses. Baia et al. showed that there was a spontaneous antibody response to NY-ESO-1 in one patient of their series (78). Additionally, they reported a correlation between grade and outcomes and NY-ESO-1 expression. There are several prospects and clinical trials testing NY-ESO-1 based immunotherapies for cancer (101). Given the beneficial results of allogeneic NK infusions in other solid tumors, it is reasonable to study this alternative for meningiomas. A study evaluated the role of NK cell infusions in addition to checkpoint blockade in a preclinical model of meningioma. This study demonstrated added and robust antitumor response in the subjects treated with checkpoint blockade and NK cell infusions (97). Other approaches including neoadjuvant immunotherapy targeting any of these molecules, alone or in combination, may be adopted for GBM patients, where neo-adjuvant anti-PD1 immunotherapy resulted in improved survival (102).

In contrast to utilizing monoclonal antibodies, which could have differing effects based on levels of immunosuppression in the tumor immune microenvironment, harnessing CAR-T cells might also present a promising treatment strategy. Recently, Tang et al. report their experience with low-dose B7-H3 targeting CAR-T infusions in a single patient through an Ommoya port (103). In response to therapy, the patient had no major adverse events and the CSF showed increased levels of various cytokines (103). However, there were no definitive signs of tumor regression on imaging (103). Additional trials exploring adequate dosing and differing targets are needed to make conclusions on the potential of CAR-T therapy for refractory meningiomas.

## Conclusion and Future Perspectives

Meningiomas are mostly benign tumors that arise from the arachnoid cells. For HGMs, complete surgical resection is not always feasible and is not curative, given that these tumors often recur and invade important structures. One of the few effective adjuvant treatment strategies available for recurrent WHO grade II and III meningiomas is radiotherapy. Chemotherapy and other pharmacologic therapies have not been effective to treat recurrent and aggressive tumors. The data presented above suggests that immunotherapy may be a good therapeutic approach for patients with difficult to treat lesions. Increasing understanding of the mechanisms used by cancer to evade immune surveillance continues to shed light on potential immunotherapeutic targets. Reversing the immunosuppressive changes produced by tumors has led to significant anti-tumor immune responses in such tumors as melanoma and lung cancer. Ultimately, a multi-target approach harnessing various components of the immune microenvironment may lead to positive results for patients with HGMs. Clinical trials involving checkpoint blockade, activation of other components of the immune system, genomic characterization, and classifications of the tumors, and careful patient selection will hopefully lead to discoveries of effective treatments for patients with aggressive meningiomas.

## Author Contributions

TG-M and EP conceived the manuscript and critically reviewed the article. DB, MP, and TG-M review of the literature. TG-M, EP, DB, and MP drafted the manuscript. All authors approved the final version of the manuscript.

## Conflict of Interest

The authors declare that the research was conducted in the absence of any commercial or financial relationships that could be construed as a potential conflict of interest.
